# Surgical treatment of Peyronie’s disease with autologous tunica vaginalis of testis

**DOI:** 10.1186/s12894-016-0120-3

**Published:** 2016-01-13

**Authors:** Bianjiang Liu, Quan Li, Gong Cheng, Ninghong Song, Min Gu, Zengjun Wang

**Affiliations:** Department of Urology, The First Affiliated Hospital of Nanjing Medical University, Nanjing, 210029 China; Department of Urology, Suzhou Municipal Hospital, Suzhou, 215000 China

**Keywords:** Peyronie’s disease, Tunica vaginalis, Autologous, Sexual function

## Abstract

**Background:**

To investigate the feasibility and safety of surgical treatment for Peyronie’s disease (PD) by excising and repairing plaque using autologous tunica vaginalis of testis.

**Methods:**

From March 2007 to December 2012, total 19 patients with PD underwent surgical treatment at our center. All patients had significant phallocampsis during erection. All patients complained of decreased sexual function. During the operation, the fibrotic plaque was excised and neurovascular bundle (NVB) was spared. A size-matching autologous tunica vaginalis of testis was harvested as the graft and patched to the defect. All patients received follow up every 3 months in the first year and 6 months in the following years. Data on sexual function before and after the operation was collected and compared.

**Results:**

All operations were completed successfully without serious complications. The mean operative time was 74 min. The mean size of excised plaque was 3.0 cm^2^. Postoperative pathological studies revealed the fibroplastic hyperplasia of excised tissue. All patients had satisfactory correction of penile appearance. The erectile penile length between pre- and post-operation didn’t show significant difference. Postoperative intercourse satisfaction and overall satisfaction measured by IIEF-5 were significant improved.

**Conclusions:**

Our surgical treatment is feasible and safe for patients with PD. It can effectively improve the penile cosmetic appearance and patients’ intercourse/overall satisfaction on sexual life.

## Background

Peyronie’s disease (PD) is a progressive fibrotic tissue disorder of the penile tunica albuginea. PD can lead to the formation of fibrous plaques, penile deformity, painful erection, loss of penile flexibility, and finally sexual dysfunction [1]. It is generally recognized that PD contains two inflammatory phase: acute and chronic phase. Medical therapy is used for the acute phase or those unfit for surgery [2]. Surgical treatment is the gold standard for PD due to the most reliable and sustained correction of phallocampsis. There are many surgical options for PD such as Nesbit procedure, Yachia procedure, Plication procedures, and grafting procedures. Grafting procedures are the focus of recent studies for effectively preserving penile length. Three types of graft have been reported: autologous graft, xenograft and synthetic graft [3]. Considering the higher rate of infection, regional inflammation reaction and allergic reaction, synthetic graft is not the main stream [4]. A variety of autologous grafts have been used including vein wall, rectus sheath, and buccal mucosa. The testicular tunica vaginalis was first reported as autologous graft in 1980. However, the technique has not been widely applied [5]. In present study, we reported our experience of surgical repair on 19 patients with PD using autologous tunica vaginalis of testis. The aim of the study was to investigate the feasibility and safety of our surgical treatment for PD.

## Methods

Approval for the study was granted by the ethics committee of Nanjing Medical University (China) and informed written consent was received from patients, including acquiring essential medical images for publication.

### Patients

From March 2007 to December 2012, total 19 patients with PD were recruited to our center. The mean age of patients was 40 (32-61) years. All patients had phallocampsis during erection. Preoperative measurement by goniometer revealed that the mean penile curvature was 36° (25–50°). All patients complained of decreased sexual function. Physical examinations revealed 11 plaques at the dorsal penis, 3 plaques at the lateral penis, and 5 plaques at the ventral penis. Surgical indications included in the stable history at least for 12 months, significant phallocampsis, and decreased erectile function related to PD after ineffective medical treatment.

### Procedures

**Fig. 1:**
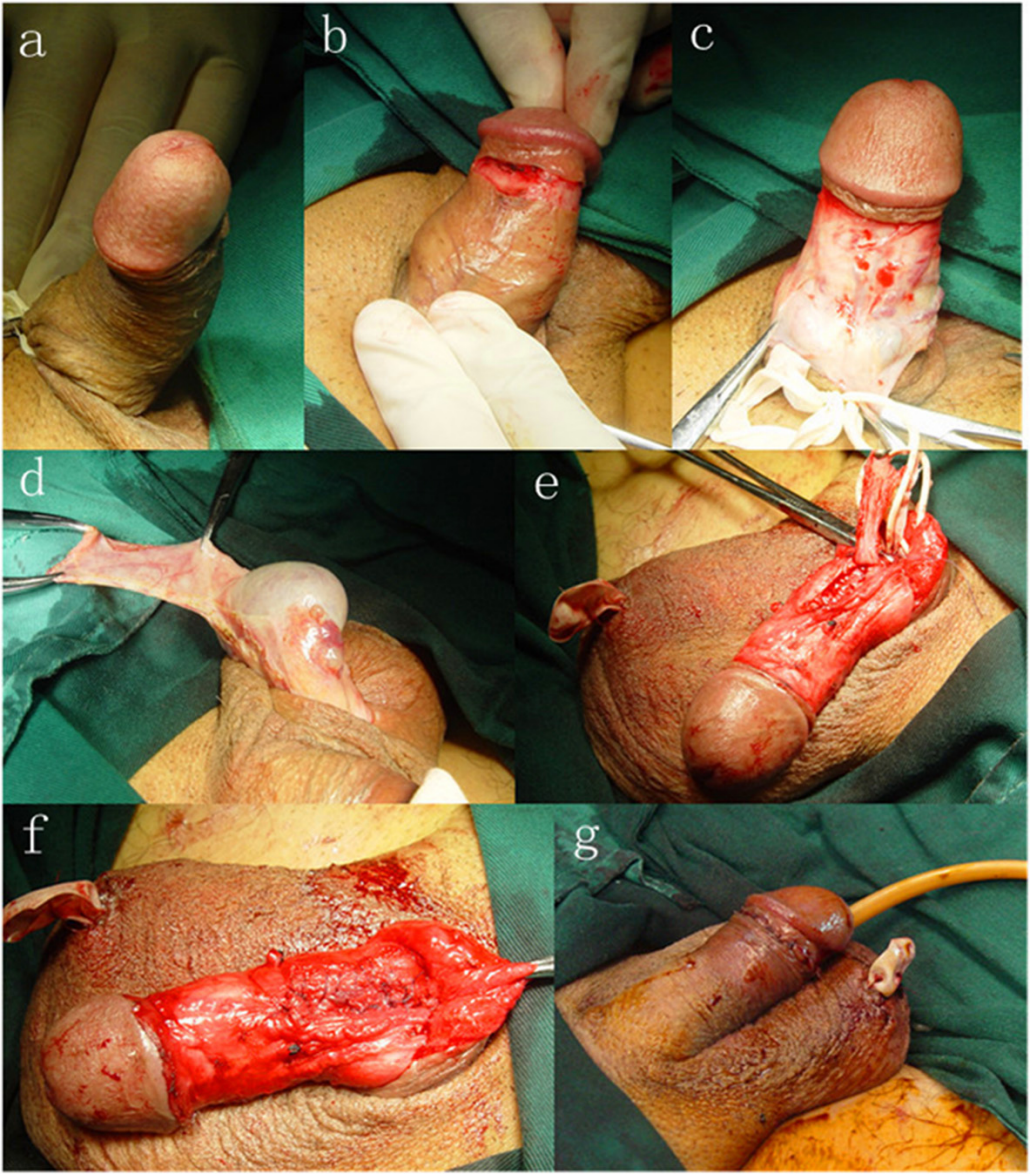
The main surgical steps of repairing PD using autologous tunica vaginalis of testis. **a** Dorsal curvature of penis. **b** Circumcision proximal to the glans coronal. **c** Degloving of penile skin to the base of the penis. **d** Harvesting tunica vaginalis of testius. **e** Suturing the flap to the albugineous defect. **f** Completion of defect repairing. **g** suturing the penile and testicular incisions and indwelling routinely the drainage tube and Foley catheter

All operations were performed by the same experienced urologists at our center. After induction of general anesthesia, the patients were placed in the supine position. A tourniquet was secured at the base of penis (Fig. 1a). A circumferential skin incision was made along the coronary sulcus to deglove penile shaft to the base of penis (Fig. 1b and c). The deep dorsal vein was isolated and clipped at both arms of the curvature. The ligated segmental vein was transected and removed. Then meticulous dissection was performed to preserve the neurovascular bundle (NVB) and to fully expose the foci on the tunica albuginea. A transverse incision was made to cut off the proximal fibrous plaque. Two longitudinal incisions were made along its bilateral sides. From the proximal end, the plague was excised deeply into the whole albugineous wall till the distal end. When the plaque was removed, the curvature could be corrected with no significant dorsal tension. The size of the defect was measured. A 2–3 cm longitudinal incision was made at the anterior wall of the scrotum. The parietal wall of tunica vaginalis was exposed for entry of the tunica cavity. To obtain a sufficient size of the graft, a rectangle tunica flap was harvested along the epididymal side (Fig. 1d). The flap was trimmed for suitable length and width to cover the defect. Then the flap and normal tunica albuginea were sutured together with 5-0 absorbable sutures (Fig. 1e and f). Artificial erection with saline injection was performed to confirm the satisfactory correction of phallocampsis. Lastly, the incisions were sutured with indwelling routinely the drainage tube and Foley catheter (Fig. 1g). During suturing penile incision, the relative position of penile shaft and degloved prepuce were determined carefully to avoid penile rotation. After operation, oral antibacterial was used. The drainage tube and catheter were removed within 24 h.

### Outcomes analysis

During the follow up, all patients received interview and physical examination every 3 months in the first year and 6 months in the following years. The International Index of Erectile Function (IIEF-5) questionnaire was made at 6 months postoperatively. Data on sexual function before and after operation was collected and compared. Data were expressed as mean ± SD. Paired *T*-test was performed for the comparison of pre- and post-opersation. Statistical analysis was made using SPSS 17.0 (SPSS Inc., Chicago, IL, U.S.). A p value < 0.05 was considered statistically significant.

## Results

Demographic and preoperative clinical characteristics of 19 patients were presented in Table 1. All operations were completed successfully without serious complications. The mean operative time was 74 (55–100) min. The mean size of excised plaque was 3.0 (1.7–4.5) cm^2^. Postoperative pathological studies revealed the fibroplastic hyperplasia of excised tissue. All patients had satisfactory correction of phallocampsis. The erectile penile length between pre- and post-operation didn’t show significant difference (11.23 ± 2.32 cm vs 11.34 ± 2.20 cm; *p* = 0.21). During 12-43 months follow up, all patients had no abnormal penile appearance and recurrent fibrous plaque.

**Table 1:** Demographic and preoperative clinical characteristics of 19 patients

Variable	Mean (range)
Age (year)	40 (32–61)
BMI	24.3 (21.1–26.7)
History of disease (year)	2.8 (1–5)
Affected location	
Dorsal	11
Lateral	3
Ventral	5
Curvature	36° (25–50°)

To explore the surgical influence on sexual function, we quantified and compared different aspects of sexual function using IIEF-5 questionnaire between pre- and post-operation (Table 2). Although the postoperative scores of erectile function, orgasmic function, and sexual desire improved, there had no significant differences compared with the preoperative status (*p* = 0.88, 0.64, and 0.13, respectively). However, the postoperative scores of intercourse satisfaction and overall satisfaction improved significantly than pre-operation (*p* = 0.02 and 0.007, respectively).

**Table 2:** IIEF-5 scores of sexual function before and after operation

	Pre-operation	Post-operation	*p* value
Erectile function	18.6 ± 3.1	18.7 ± 2.2	0.88
Orgasmic function	7.5 ± 1.0	7.6 ± 1.0	0.64
Sexual desire	8.0 ± 0.9	8.5 ± 1.1	0.13
Intercourse satisfaction	7.7 ± 1.1	9.0 ± 2.0	0.02*
Overall satisfaction	6.5 ± 1.1	7.2 ± 0.9	0.007*

Data were shown as Mean ± SD

*p value < 0.05

## Discussion

Surgical treatment for PD is necessary if the patients had significant phallocampsis, decreased sexual function related to PD, or ineffective medical treatment. Three surgical methods can be used for PD: penile tunica albuginea placation, grafting procedures, and penile prosthesis implantation [6–8]. Tunica albuginea placation is a relatively simple surgical procedure. However, it may lead to postoperative penile shortening. Penile prosthesis implantation is technically complicated and expensive. Grafting procedures are now the focus of surgical treatment for PD. The material selection is currently controversial. Autologous graft, xenograft and synthetic graft can be used [4, 9, 10]. The ideal graft material should be readily available, pliable, inexpensive, resistant to infection and able to preserve erectile function. Autologous graft are most common used due to their easy incorporation into host tissue and few incidence of local inflammatory reaction [11, 12].

In current study, we chose autologous tunica vaginalis of testis for repairing PD. The first is that tunica vaginalis is relatively superficial and convenient to harvest. It has good blood supply and histocompatibility after transplanting. Compared with synthetic graft, the autologous graft is more economical and has lower risk of graft removal due to postoperative infection [13, 14]. Second, tunica vaginalis has uniform thickness and good pliability and elasticity to guarantee the penile erection. In present study, we observed that the tunica vaginalis had a good viability and satisfactory function exertion. Furthermore, tunica vaginalis incision is more safe and simple than other autologous graft such as vein wall, rectus sheath, and buccal mucosa. No significant surface scars, pains, and regional complications occurred after operation. During the follow up, all patients had no recurrence of fibrotic plaque or curvature at the graft site. No recurrent phallocampsis during the erection was observed. The correction of penile deformity during erection promoted the sexual quality and correspondent sexual satisfaction. Only one patients thought that the surgery bring slight shortening of penile length despite the curvature had been corrected. Our data showed the short- and mid-term effectiveness of autologous graft. Most significant benefits for patients receiving surgery were the improvement on penile appearance and sexual satisfaction.

ED is a well-recognized co-morbidity of PD. PD, due to penile deformity, may make intercourse less enjoyable, more awkward, and even impossible [15]. Although improving the sexual function, the surgery could also lead to de novo ED [6]. The possible causes of postoperative ED include in the progression or recurrence of the disease, injury of NVB and psychological influence. In our study, 79 % of patients had their erectile function moderately affected. After operation, the sexual function was improved generally. Although there were no significant differences between preoperative and postoperative scores of erectile function, orgasmic function, and sexual desire, postoperative intercourse satisfaction and overall satisfaction were improved obviously. The results should be attributed to the correction of penile deformity and enhancement of patients’ self-confidence. There is no single parameter or combination of medical comorbidities to adequately predict the development of ED after PD. Strictly complying with surgical rules, careful pre-operative counseling, and post-operative physiological caring are essential to decrease de novo ED. There was no postoperative ED in our study. The result confirmed the safety of our method in treating PD.

One limitation of our study includes its retrospective design. We can’t compare with the clinical outcomes between tunica vaginalis and autologous grafts or xenografts. In addition, although the short- and mid-term outcomes of our surgical treatment are good, the long-term outcomes, especially the influence on the sexual function, remain to be seen.

## Conclusions

Our surgical treatment is feasible and safe for patients with PD. It can effectively improve the penile cosmetic appearance and patients’ intercourse/overall satisfaction on sexual life.

## Abbreviations

IIEF-5: International Index of Erectile Function
NVB: neurovascular bundle
PD: Peyronie’s disease
